# Evaluation of Chitosan Derivative Microparticles Encapsulating Superparamagnetic Iron Oxide and Doxorubicin as a pH-Sensitive Delivery Carrier in Hepatic Carcinoma Treatment: An *in vitro* Comparison Study

**DOI:** 10.3389/fphar.2018.01025

**Published:** 2018-09-21

**Authors:** Meng-Yi Bai, Sung-Ling Tang, Meng-Han Chuang, Ting-Ying Wang, Po-da Hong

**Affiliations:** ^1^Graduate Institute of Biomedical Engineering, National Taiwan University of Science and Technology, Taipei, Taiwan; ^2^Biomedical Engineering Program, Graduate Institute of Applied Science and Technology, National Taiwan University of Science and Technology, Taipei, Taiwan; ^3^Adjunct appointment to the Department of Biomedical Engineering, National Defense Medical Center, Taipei, Taiwan; ^4^Global Taiwan Center for Excellence for Thin-Film Metallic Glass, Taipei, Taiwan; ^5^Department of Pharmacy Practice, Tri-Service General Hospital, Taipei, Taiwan; ^6^Department of Materials Science and Engineering, National Taiwan University of Science and Technology, Taipei, Taiwan

**Keywords:** nanotechnology, doxorubicin, superparamagnetic iron oxide, electrospray, microparticle

## Abstract

We developed a novel, pH-sensitive drug delivery microparticle based on *N-*palmitoyl chitosan (NPCS) to transport the superparamagnetic iron oxide (SPIO) and anticancer drug doxorubicin (DOX). The characteristics of NPCS were characterized through nuclear magnetic resonance. Our results based on testing of volume swelling in multiple pH aqueous solutions revealed that the modified chitosan had a pH-sensitive property. The morphology and size of the DOX-SPIO/NPCS microparticles were investigated using transmission electron microscopy and scanning electron microscopy. The statistical result of microparticles had diameter of 185 ± 87 nm. Surface chemical moieties of DOX-SPIO/NPCS microparticles were confirmed using attenuated total reflection Fourier transform infrared spectroscopy and indicated the existence of mostly hydrophilic groups such as -OH, -C=O, and -C-O-C-. Transmission electron microscopy revealed the dark contrast of SPIO dots encapsulated in the NPCS matrix. Nuclear magnetic resonance T2-weighted magnetic resonance imaging confirmed that the produced DOX-SPIO/NPCS microparticles still exhibited T2 relaxation durations as short as 37.68 ± 8.69 ms (under administration of 2.5 μg/mL), which is comparable to the clinically required dosage. In the drug release profile, the DOX-SPIO/NPCS drug delivery microparticle was accelerated in an acidic environment (pH 6.5) compared with that in a basic environment. Microparticles in a cytotoxicity assay (3-[4,5-dimethylthiazol-2-yl]-2,5-diphenyltetrazolium bromide assay) revealed that DOX-SPIO/NPCS microparticles had better antitumor ability than did free-form of DOX. Additionally, microparticles loaded with 0.5–5 μg/mL DOX in an acidic environment (pH 6.5) demonstrated higher efficacy against Hep G2 cell growth, possibly because of the swelling effect of NPCS, resulting in volume expansion and easy drug release. Accordingly, these large DOX-SPIO/NPCS microparticles showed potential for application as a pH-sensitive drug delivery system and as chemoembolization particles for hepatic carcinoma therapy.

## Introduction

Hepatocellular carcinoma (HCC) is the third leading cause of cancer-related death in the world, and the incidence of HCC is still increasing in the United States and Canada (Ayuso et al., 2018). Transcatheter arterial chemoembolization (TACE) has become the current standard therapy for unresectable HCC because systemic chemotherapy is ineffective and surgery is feasible for only 15–30% of patients with HCC (Ramsey and Geschwind, 2002; Hsu et al., 2004). TACE involves precise navigation of a catheter into an arterial branch that feeds mainly the hypervascular tumor but none of the surrounding healthy tissue. Subsequently, micrometer-level polymeric particles are injected via the catheter, and arterial blood flow carries them into the tumor site. Because of the branching structure of angiogenesis, the embolic particles become stuck in the tumor's arterial vessel bed, and consequently, tumor tissue behind each obstruction is no longer perfused; this leads to ischemia and necrosis in these parts of the tumor tissue.

Currently commercially available embolic materials include metallic coils, oils (Lipiodol), non-spherical particles (Gelfoam; Pfizer, New York, NY, USA), and microspheres (Embosphere and HepaSphere, Merit Medical Systems, South Jordan, UT, USA; DC Beads, BTG International, London, UK) (Weng et al., 2013). However, TACE has some clear disadvantages with respect to HCC treatment; the creation of ischemic conditions at the tumor site promotes a proangiogenic response that enables the tumor to continue to thrive. Furthermore, two popular embolic materials—the DC Bead and HepaSphere—are not biodegradable, and long-term presence of DC Bead microspheres in the body may induce inflammation, thereby causing further tissue injury (Liu et al., 2015). In addition, the permanency of the microspheres limits the opportunity for the vessel recanalization required for repeating TACE procedures, and the drug release measured *in vitro* is incomplete for doxorubicin (DOX) loaded on the beads (Jordan et al., 2010). Moreover, complete necrosis of liver tumors is very difficult to achieve with TACE alone, and the long-term prognosis is unsatisfactory (Oliveri et al., 2011). Evidence suggests that TACE combined with other therapies such as radiotherapy or radiofrequency ablation for treatment of HCC can produce therapeutic benefits and have a synergistic effect.

To overcome the aforementioned problems, in this study, we focused on developing a new type of spherical, biodegradable, therapeutically synergistic, and drug-loadable embolic material that possesses bimodality to improve the efficacy of tumor embolization. Chitosan (CS), a natural origin polysaccharide, is biodegradable, nontoxic, and compatible with soft tissue, and thus can be formulated with a chemotherapeutic agent (DOX) and a hyperthermia agent (superparamagnetic iron oxide [SPIO]) to create a combination of candidate materials to construct a microsphere for drug delivery and imaging. Our previous study demonstrated that DOX alongside CS microparticles with encapsulated SPIO as a hyperthermia agent is effective for hepatic cancer treatment (Tang et al., 2017). However, this drug delivery vehicle lacks selectivity with respect to killing cancerous tissue; that is, it is unable to target cancerous tissue without harming healthy tissue.

Cancer tissue increases metabolic activity and hypoxia, resulting in elevated extracellular acidity (Stubbs et al., 2000). Therefore, CS has been selected and modified to change its properties and enable it to respond in an acidic environment; many CS derivatives have been developed in this manner (Jiang et al., 2006; Liang et al., 2007). Evidence suggests that incorporation of hydrophobic chains into the CS structure could markedly accelerate drug release when the pH is low. Thus, in the present study, to improve the selectivity of drug release at targeted sites and examine the microspheres' pH-sensitive ability (Jiang et al., 2006), we conjugated a hydrophobic palmitoyl group onto the free amine groups of CS to generate the N-palmitoyl chitosan (NPCS) in a one-step synthesis procedure, which follows previous studies but with slight modification (Chiu et al., 2009; Chen et al., 2011). To the best of our knowledge, no studies have reported using NPCS as an embolic material with encapsulation of DOX for drug delivery through the hepatic artery. The aim of this study was to present a proof-of-concept evaluation of the synthesis, characterization, and preliminary drug delivery of DOX-SPIO/NPCS microparticles that simultaneously combines NPCS, DOX, and magnetic material like iron oxide particles in a single microparticle; the objective was to exploit these vehicles' potential as a drug delivery system for liver cancer following transcatheter embolotherapy that is capable of providing bimodal chemoembolization and hyperthermia. This multimodality treatment is anticipated to prevent side effects in some patients such as poor response to systemic chemotherapy and inability to receive surgery.

## Materials and methods

### Materials

Doxorubicin HCl (DOX) was obtained from TTY Biopharm (Taipei, Taiwan) as a gift. CS (viscosity, 5 g/L; degree of deacetylation, 80.0%) was purchased from Wako Pure Chemical Industries, Ltd. (Osaka, Japan). Palmitic acid *N*-hydroxysuccinimide ester was bought from Sigma Aldrich Corp (Sigma Aldrich, MO, USA). SPIO suspension was received from Taiwan Advanced Nanotech (Taoyuan, Taiwan). Dulbecco's modified Eagle's medium (DMEM), antibiotic solution (100 IU/mL penicillin and 100 μg/mL streptomycin), fetal bovine serum (FBS), and pH 7.4 phosphate-buffered saline (PBS) were supplied by Thermo Fisher Scientific (Carlsbad, CA, USA). Glacial acetic acid was purchased from Merck (Darmstadt, Germany). All other chemicals were of reagent or tissue culture grade.

### Synthesis and characterization of NPCS

**Scheme 1:**
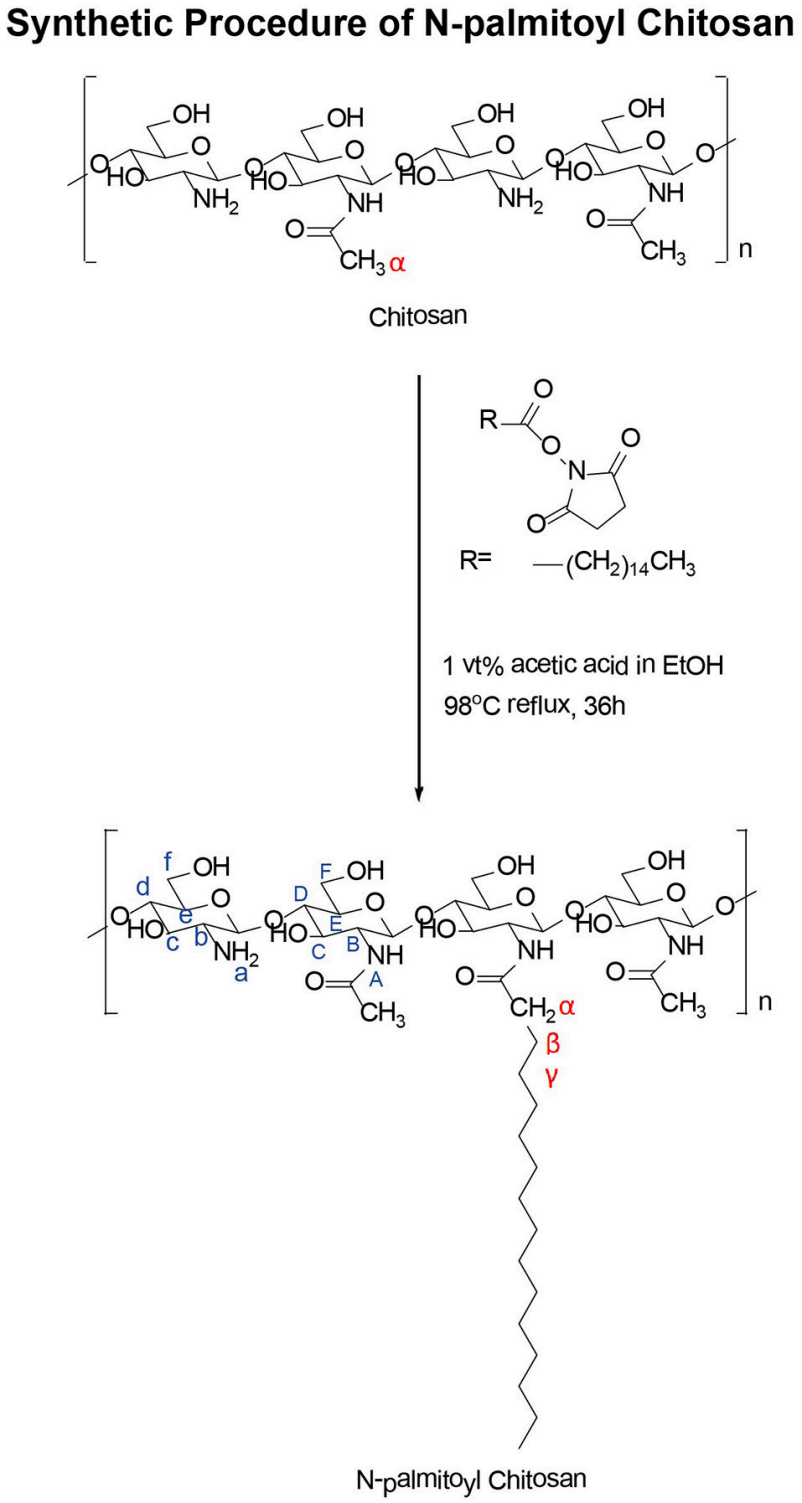
Synthetic procedure of N-palmitoyl chitosan.

Synthesis of NPCS was performed as described in Scheme 1. In summary, 1.0 g of CS was dissolved in aqueous solution of 1% (vol/vol) acetic acid (1 mL of glacial acetic acid mixed with 100 mL of deionized water) solution. This solution was followed by adjustment with 14.5 mL of 1 N NaOH solution until the pH reached 6. Next, 0.3 g of palmitic acid *N-*hydroxysuccinimide ester was dissolved in 20 mL of 99% alcohol to create a reactant stock solution. This stock solution was added dropwise to the CS aqueous solution and allowed to reflux at 98°C for 36 h. NPCS was obtained by adding acetone to the reaction mixture for precipitation. Filtration and washing were then performed to harvest and purify the educts. The product was dried in an oven for subsequent characterization and future use.

Nuclear magnetic resonance (NMR) spectroscopy was used to characterize the chemical structure of NPCS. NPCS powder samples (1 wt%) were dissolved in d-formic acid. ^1^H-NMR spectra were acquired on an AV-400 MHz spectrometer (Agilent Technologies, Karlsruhe, Germany).

### Swelling tests of NPCS

NPCS gels were prepared by stirring 0.1 g of dry NPCS powder in 3 mL of 10% acetic acid aqueous solution at room temperature for 24 h; the gel-like semiliquid produced was poured into a syringe tube. Subsequently, this NPCS gel was protruded and transformed into droplets onto a petri dish, before 15 mL of pH 6.5 or 7.4 distilled water were separately added to each dish to entirely cover the gels. At a predetermined time-interval, the samples were photographed using a digital camera, and their areas and volume were determined using ImageJ software (National Institutes of Health, Bethesda, MD, USA).

### Preparation of DOX-SPIO/NPCS microparticles

We set up an electrospray (ES) system as described in our previous studies (Lee et al., 2010; Bai and Yang, 2013; Bai and Liu, 2014; Lin et al., 2016; Tang et al., 2017). First, 50 mg of NPCS was dissolved in 2 mL of formic acid and stirred overnight to generate NPCS solution. Subsequently, 1 mL of acetone was added to the NPCS solution and 5 mg of DOX was then fully dissolved in the mixture under magnetic stirring. Finally, 1 mL of SPIO was added dropwise to the mixture to generate the DOX-SPIO/NPCS stock solution, which was delivered using a syringe pump (NE-300 Just Infusion™; New Era Pump Systems, Farmingdale, NY, USA). A 20-gauge flat-tipped needle was used as the capillary tube in this experiment. A positive voltage was applied to the spray head assembly by a direct current high-voltage power supply (Bertan Model 205B-20R; Spellman High Voltage Electronics, Hauppauge, NY, USA) to establish a spray electrical field between the capillary tube and the electrically grounded collection substrate. The collection substrate in this setup could be a metal plate or distilled water, depending on the purpose of the application. A capillary tube was placed approximately 6.5 cm high over the collection substrate. The applied voltage ranged from 10 to 17 kV. The ES modes of the system were monitored by viewing the liquid meniscus at the exit of the capillary tube. In addition, the meniscus was illuminated with diffuse light provided by an optical cable light, and its droplet shape was observed using a microscopic system comprising a microscope lens (model 30-43-10, Optem RetroZoom 65; Rhyl, UK), a digital camera (model STC-620PWT; Sentech, Carrollton, TX, USA), and a high-resolution liquid crystal display monitor. Finally, the obtained DOX-SPIO/NPCS microparticles were collected by discharging the supernatant after centrifugation. The amount of total free DOX was determined through spectrophotometry by using a SPECTROstar Nano instrument (BMG LABTECH, Ortenberg, Germany).

### Characterizations of DOX-SPIO/NPCS microparticles

#### Morphology and structural characterization of microparticles

The morphology of the particles was inspected through scanning electron microscopy (SEM) under an accelerating voltage of 20 kV (JSM-6390L; JEOL, Tokyo, Japan). To prevent charge accumulation, all SEM sample specimens were coated with an additional thin layer of platinum film (sputtering time, 90–110 s). For transmission electron microscopy (TEM), the DOX-SPIO/NPCS microparticle suspension was placed on a formvar/carbon film-coated copper grid (Ted Pella, Redding, CA, USA). After the excessive sample had been removed with filter paper, the copper grid was air-dried at room temperature. The specimens were then imaged using a transmission electron microscope (JEM-1230; JEOL). The DOX concentration in DOX-SPIO/NPCS microparticle suspensions was determined using a spectrophotometer (SPECTROstar Nano). First, the absorbance spectra of DOX were recorded at scanning wavelengths falling between 300 and 800 nm to obtain data on the maximum absorption wavelength of 480 nm. A calibration curve was established by plotting the absorption value of DOX at 480 nm against a series of predetermined concentrations of standard solutions. Moreover, the attenuated total reflectance infrared (ATR-IR) spectra of the freeze-dried DOX-SPIO/NPCS microparticles were acquired using a Fourier transform infrared spectroscopy spectrometer (FT/IR-4680; JASCO, Easton, MD, USA) equipped with an attenuated total reflectance accessory. All ATR-IR spectra were acquired from 36 cycles of accumulation to increase the signal-to-noise ratio.

#### Magnetic resonance imaging

Magnetic resonance imaging (MRI) was performed using a clinical 1.5-T MRI system (SIGNA; GE Medical Systems, Milwaukee, WI, USA) and a one-channel lower extremity coil. All images were obtained as follows: a sagittal two-dimensional fast spin echo (FSE) sequence (echo time/repetition time, 99/6000; number of excitations, 8; echo train length, 32), 192 × 192 matrix, field of view of 100 × 100 mm, and section thickness of 2 mm. The experimental parameters were referred to details provided in previous studies (Chen et al., 2015; Tang et al., 2017).

#### *In vitro* release test

A dialysis method using a molecular porous membrane (Spectra/Por 3.5-kD molecular weight cutoff; Spectrum Medical Industries, Inc., Rancho Dominguez, CA, USA) was employed to enable the free DOX released from the microparticles and passed freely through its pores. The DOX-SPIO/NPCS microparticle suspension and free-form DOX solution (1 mg/mL) were poured into dialysis bags that were then placed in beakers filled with 200 mL of distilled water at pH 6.5 or 7.4. The suspensions were then dialyzed at 37°C with a magnetic stirrer for agitation at 100 rpm. The temperature of the experimental system was set at 37°C. At a predetermined time interval, all distilled water in the beakers was removed and replaced with 200 mL of fresh distilled water at pH 6.5 or 7.4. The DOX concentration in the used distilled water was subsequently measured through absorbance at 480 nm. Finally, the cumulative percentage of drug release at a specific time interval was calculated according to the total amount of DOX loading measured at the beginning of this study.

### *In vitro* experiments

#### Cell culture

Human Hep G2 cell lines were obtained from the American Type Culture Collection (Manassas, VA, USA). Cells were cultured as previously described (Yeh et al., 2011; Hu et al., 2014), and cells at the 30th passage were used in this study. The cells were maintained as monolayer cellular cultures in Gibco Dulbecco's minimal essential medium (Life Technologies/Thermo Fisher Scientific) supplemented with 1% penicillin-streptomycin (Biological Industries, Inc., Cromwell, CT, USA), 1% nonessential amino acid (Biological Industries, Inc.), 3.75 g/L NaHCO3 (Sigma-Aldrich, St. Louis, MO, USA), and 10% FBS (Life Technologies) for the Hep G2 cells in 5% CO_2_ at 37°C.

#### *In vitro* cell viability/cytotoxicity studies toward hep G2 cell line

A tetrazolium salt 3-(4,5-dimethylthiazol-2-yl)-2,5-diphenyltetrazolium bromide (MTT) assay was performed to assess the viability of the Hep G2 cells after they had been treated with the DOX-SPIO/NPCS microparticle suspension. In the typical procedure, the Hep G2 cells were seeded onto a 24-well plate at a density of 1 × 10^5^ cells/500 μL (500 μL/well) in serum-containing DMEM for 24 h at 37°C in a 5% CO_2_ atmosphere. After 24 h, 1,000 μL of 50, 5, and 0.5 μg/mL DOX-SPIO/NPCS microparticle suspensions were added to each well for drug treatment. After another 24, 48, or 72 h of incubation at 37°C in a 5% CO_2_ atmosphere, the supernatant was removed from each well and the cells were washed with 500 μL of 1 × PBS solution. Finally, 300 μL of MTT reagent (5 mg/mL in PBS) was added to each well and the plate was subsequently incubated for 4 h until purple precipitate was visualized. Then, all supernatants were removed and replaced with 300 μL of dimethyl sulfoxide solution. The plate was covered and left in darkness for 20 min at 37°C in a 5% CO_2_ atmosphere. A spectrophotometer was used to measure the optical density (OD) of the supernatant at 570 nm. Cell viability was calculated as the ratio of the recorded OD values by using the following equation:

Cell viability = OD of the drug-treated group/OD of the medium-only group × 100%

### Statistical analyses

All data are presented as mean ± standard deviation. All statistical analyses were performed using IBM SPSS Statistics software (IBM, Armonk, NY, USA). Specifically, student's *t*-test, a one-way analysis of variance, and Wilcoxon statistics were used to assess the differences between the experimental groups. Differences with *P* values lower than 0.05 were considered statistically significant.

## Results

### Characterization of NPCS

**Figure 1:**
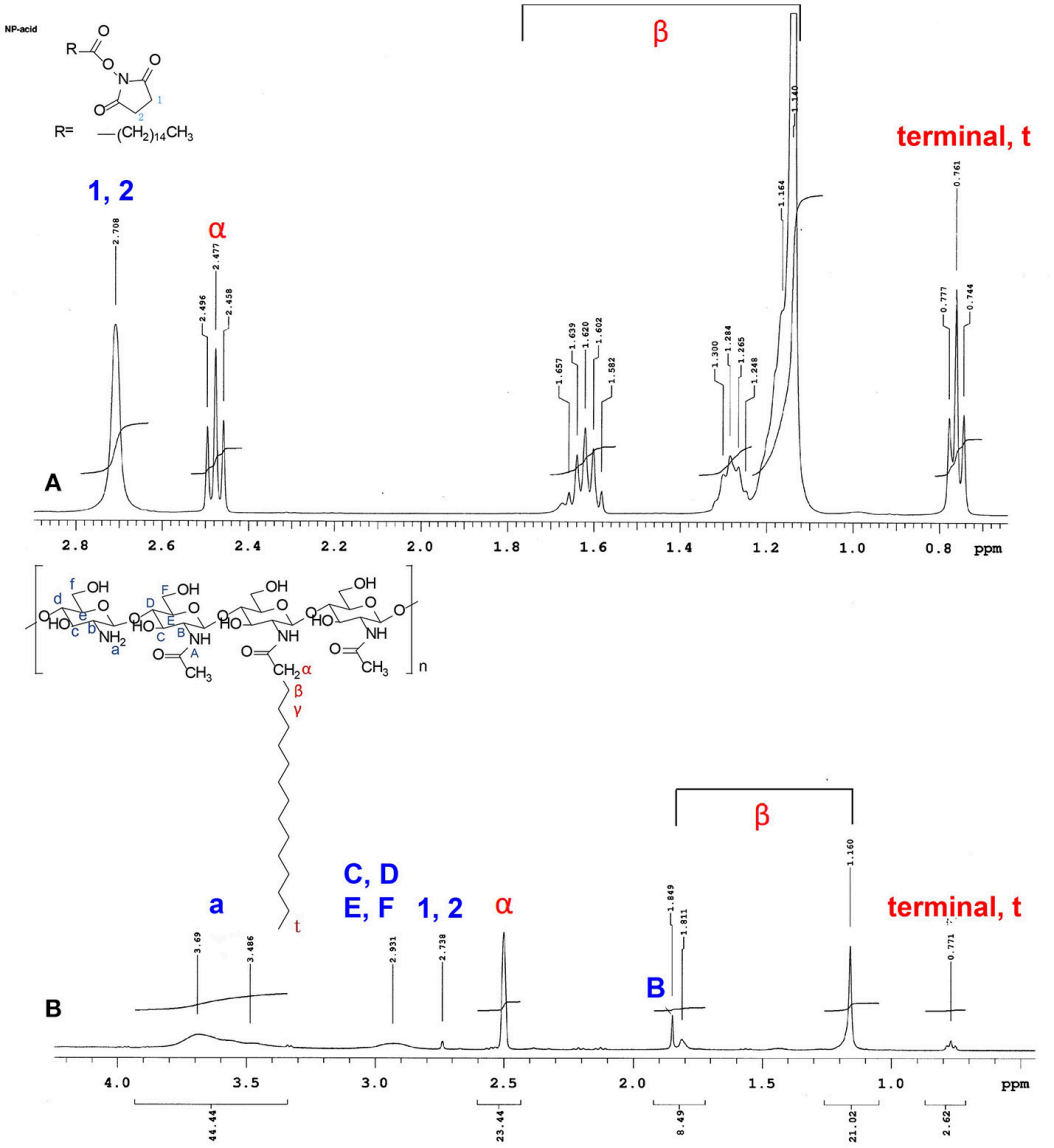
^1^H NMR spectrum of **(A)** *N*-hydroxysuccinimide ester, and **(B)** N-palmitoyl chitosan. All of the samples were dissolved formic acid-d2 (95% w/w in D_2_O).

The conjugation of palmitic acid to the CS backbone was examined through high-resolution ^1^H-NMR spectroscopy. Figure 1 displays the ^1^H-NMR spectra of palmitic acid N-hydroxysuccinimide ester (Figure 1A) and NPCS (Figure 1B). The chemical shifts for N-hydroxysuccinimide ester in d-formic acid (Figure 1A) were as follows: δ2.708 (singlet, 1, 2, succinimide), 2.477 (triplet, α, CH2), 1.140–1.657 [multiplet, β, (CH2)13], and 0.761 (triplet, terminal, CH3) ppm. The chemical shifts for NPCS in d-formic acid (Figure 1B) were as follows: δ2.500 (singlet, α, CH2), 1.160–1.849 [multiplet, β, (CH2)13], and 0.771 (triplet, terminal, CH_3_) ppm. In the NMR spectra, no peaks were observed around δ2.100, and the chemical shift of α-protons was far further downfield (Figure 1B, α-protons) than the value shown in the simulated ^1^H spectra; this illustrated that the deacetylation of CS was complete and the α-protons of conjugated *N-*palmitoyl side chain were possibly deprotonated because the α-position of protons possessed an acidic property. Therefore, downfield shifting (Δδ ≈ 0.023) of the α-position of protons on the *N-*palmitoyl chain was observed at δ2.500 as compared with the mother *N-*hydroxysuccinimide ester, which was in response to the similar chemical structure reported for *N-*terminal tyrosine (Tapia-Hernndez et al., 2015). Notably, the 1 and 2 protons on the succinimide ring at approximately δ2.708 were greatly reduced after conjugation; this indicated that the succinimide ester ring was hydrolyzed after reaction, and this leaving group could act as a conjugate base to deprotonate the α-position of protons on the *N-*palmitoyl chain. In addition, based on the following equation, 4% of *N-*palmitoyl substitution degree was estimated from the aforementioned NMR results:

$$\begin{array}{l} \text{Substitution degree}\left(\text{\%}\right)\\ \;=\left[1/3\times \text{Area C}{\text{H}}_{3\left(\text{terminal of}\text{N-palmitoyl group}\right)}\right]\\ \;/\text{Area }{\text{H}}_{2\;\left(\text{amino protons}\right)}/2\;\times \;100\text{\%}\\ \;=\left(2.62/3\right)/\left(44.44/2\right)\times 100\text{\%}\\ \;=4\text{\%} \end{array}$$

All of these changes provide evidence that the palmitoyl group was successfully grafted onto the CS backbone.

### Swelling degree of NPCS

**Figure 2:**
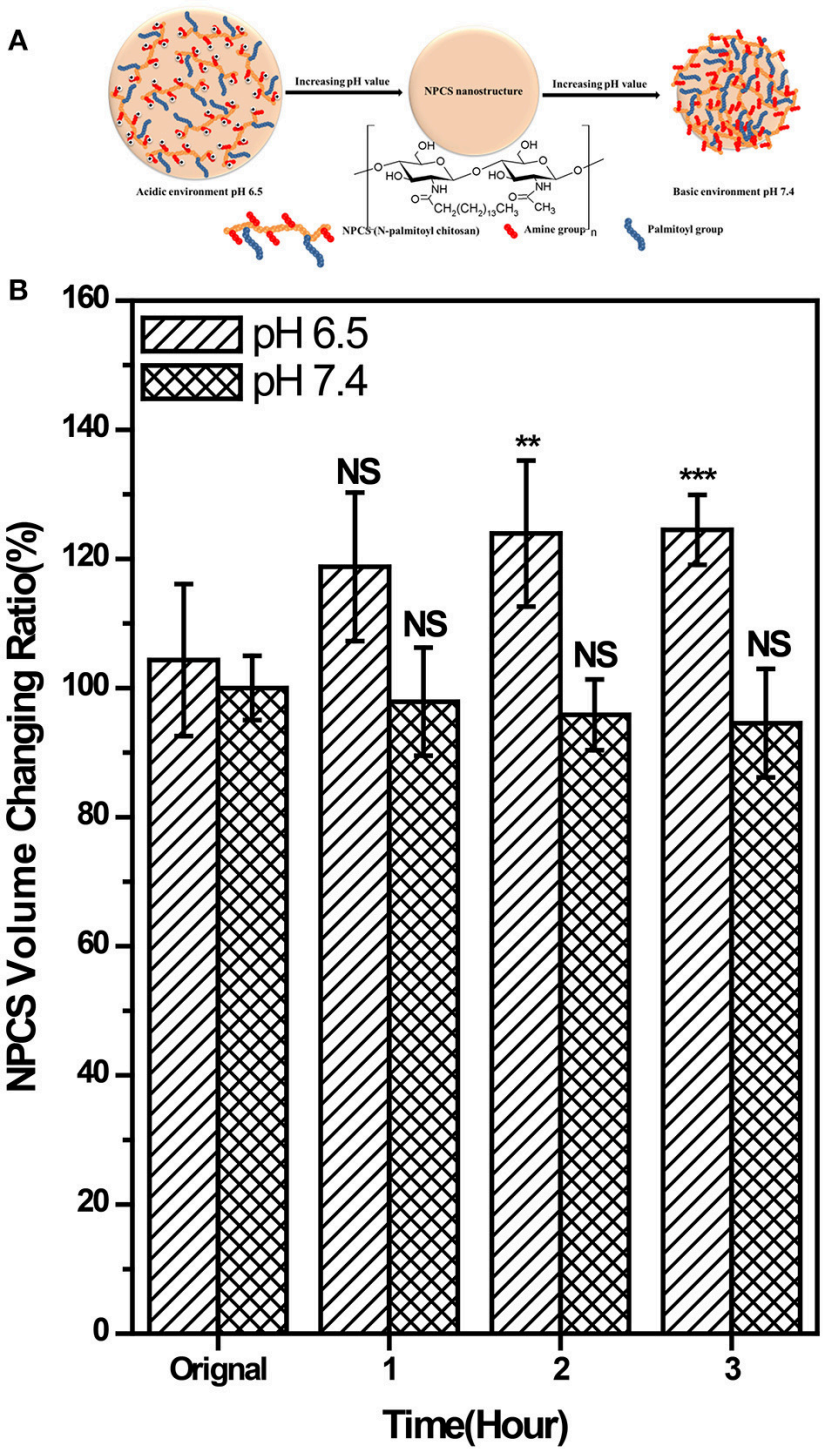
**(A)** schematic illustration of the plausible swelling mechanism of the produced DOX-SPIO/NPCS microparticle, and **(B)** pH-dependent of statistical results of volume change of NPCS gel droplets measured at different time-point. The volume changing ration can be seen as an index for evaluating the swelling degree of NPCS. **p < 0.01, and ***p < 0.001, respectively.

The NPCS area changing ratio (%) was calculated using equation:

$$\begin{array}{l} \text{changing ratio}\left(\text{\%}\right)=\left({\text{A}}_{\text{n}}\;-\;{\text{A}}_{0}\right)/{\text{A}}_{0}\;\times \;100\% \end{array}$$

in which A_0_ is the NPCS area at time 0, and An is the NPCS area at the time points of 1, 2, and 3 h, respectively (see Figure S1). The plausible swelling mechanism and results of the swelling degree of NPCS are shown in Figures 2A,B. In a pH-6.5 environment, the volume of the NPCS droplets increased by 18.7, 23.9, and 24.5% at the time points of 1, 2, and 3 h, respectively, compared with the droplets at the starting point. By contrast, the reduction in volume between 2.11 and 5.45% in a pH 7.4 environment was observed. Because the amino groups in the NPCS molecules were protonated to become ammonium ions (NH^3+^) in the acidic environment, and these positive charges in the gel phase generated electrostatic repulsive forces between polymer chains. On the other hand, when in a basic environment, deprotonation occurred and the van der Waals forces provided by the long alkyl chain became dominant forces, which inversely generated an attractive force as opposed to a repulsive force between chains. However, because the substitution degree of the N-palmitoyl chain was low in relation to the units of amino groups on the backbone of CS, a less marked change was observed in volume reduction in the basic environment than in the case of obvious volume expansion in the acidic environment. This pH response feature in the acidic environment of the produced NPCS renders this material especially suitable for selectively targeted drug delivery at tumor sites because tumor cell exhibits abnormal proliferation and generates more lactic acid and hypoxia as compared to normal tissue, resulting in elevated extracellular acidity in tumor tissue (Stubbs et al., 2000).

### Composition of resultant electrosprayed DOX-SPIO/NPCS microparticles

**Figure 3:**
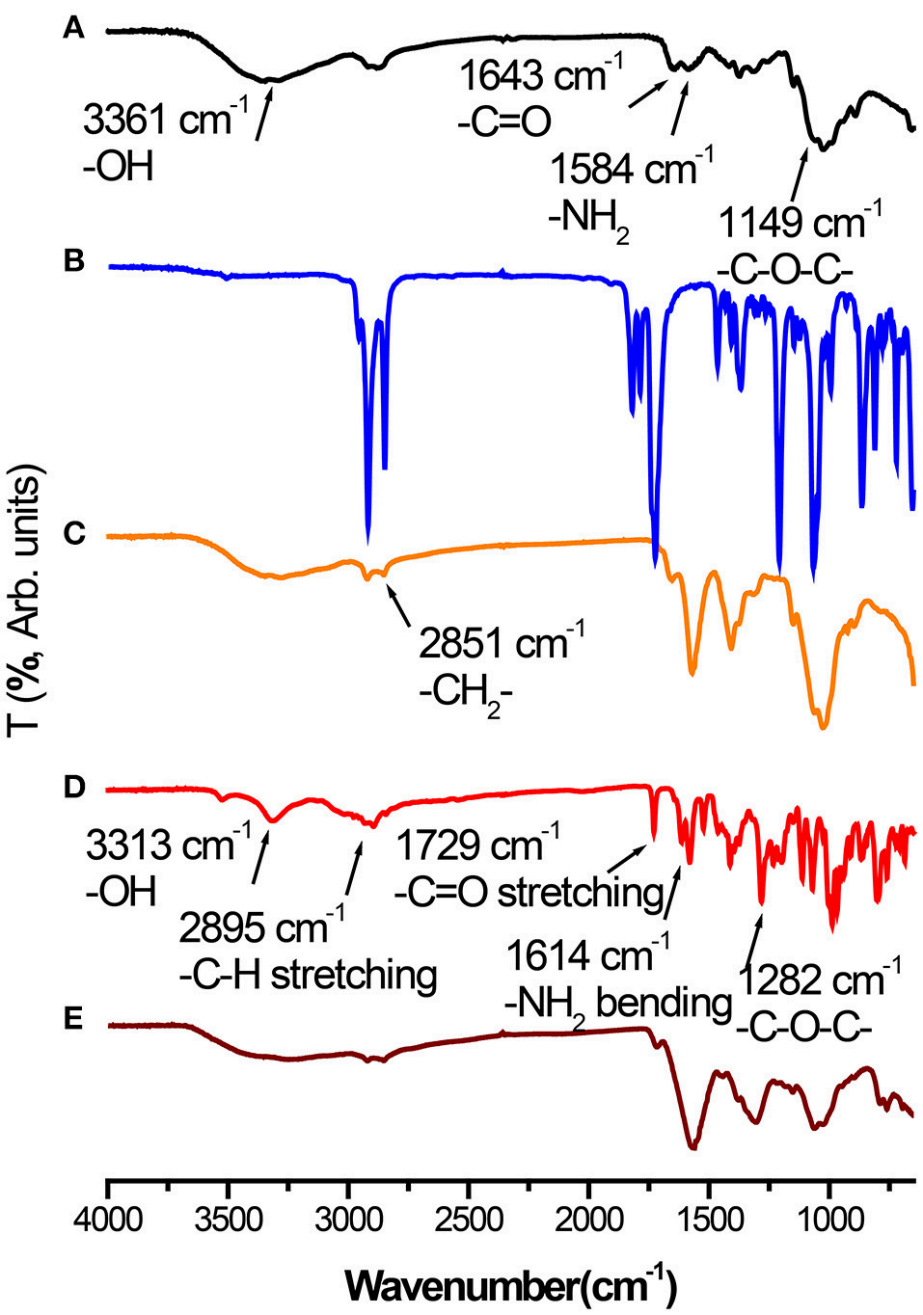
Fourier-transform infrared (FT-IR) spectra of **(A)** Chitosan powder, **(B)** palmitic acid N-hydroxysuccinimide ester powder, **(C)** NPCS powder, **(D)** DOX powder, and **(E)** DOX-SPIO/NPCS microparticles.

Fourier transform infrared (FTIR) spectra of CS (Figure 3A), palmitic acid N-hydroxysuccinimide ester (Figure 3B), NPCS (Figure 3C), DOX (Figure 3D), and DOX-SPIO/NPCS microparticles (Figure 3E) are shown in Figure 3. The peaks at 3,361, 1,643, 1,584, and 1,149 cm^−1^ represent the characteristic functional group of -OH, C = O-NHR, -NH2 (bending mode), and C-O-C groups of CS, respectively. When the amine groups of CS were conjugated with palmitoyl groups, enhancement of the peak intensity and broadening at the 2,851 cm^−1^ vibration peak were observed and attributed to the functional group of -CH2, thereby further confirming that palmitoyl groups were successfully grafted onto the CS. The spectrum of DOX-SPIO/NPCS microparticles (Figure 3E) is very similar to that of NPCS, except that several small peaks falling within 600–789 cm−1 were designated as contributions from DOX and SPIO (Cloupeau and Prunet-Foch, 1994), demonstrating that the ES process could simultaneously encapsulate multiple drugs inside the matrix material and generate drug carriers in only one step without changing its chemical property.

### Morphological and structural characterizations of microparticles using field emission SEM and TEM

**Figure 4:**
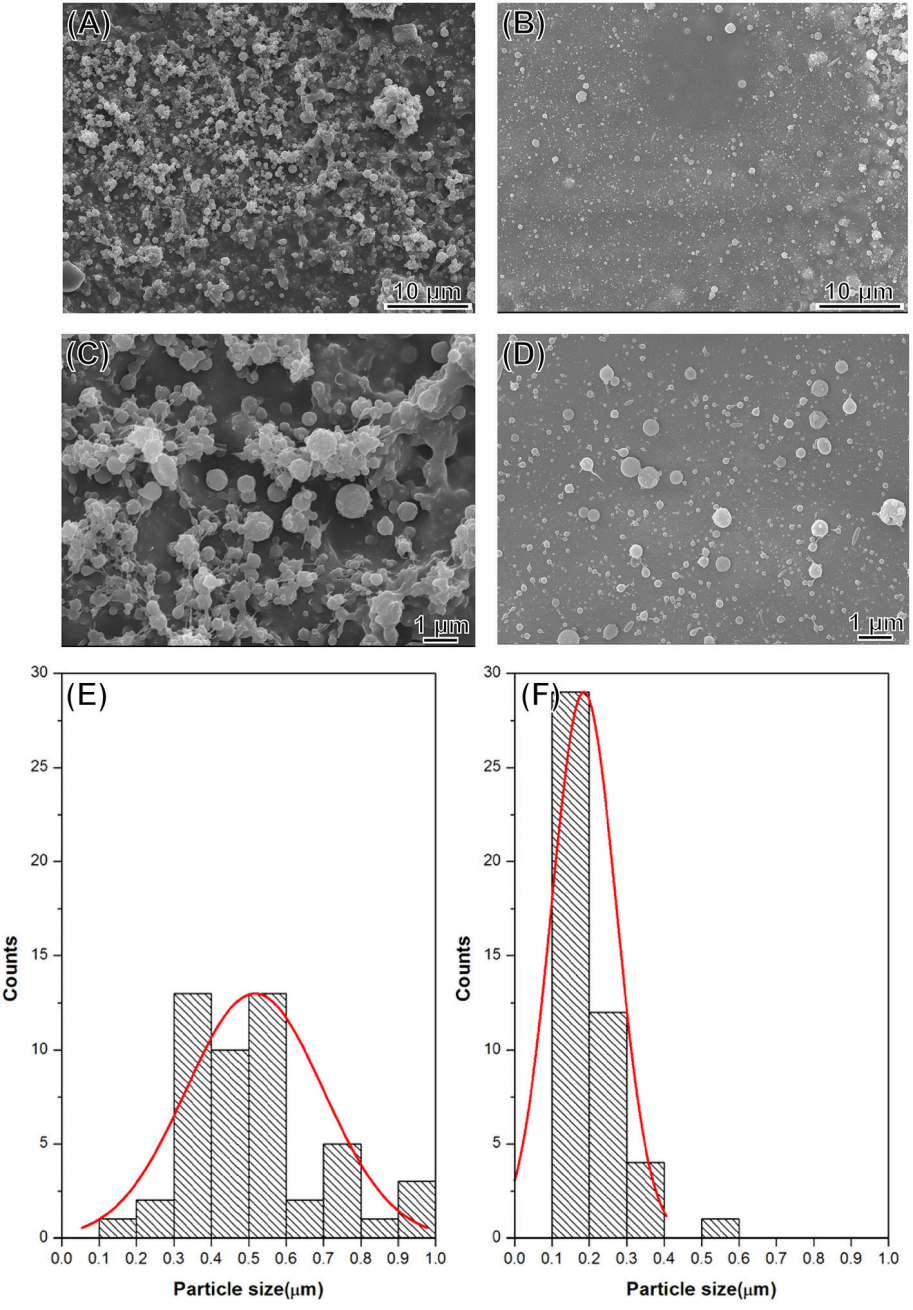
SEM images and statistic bar charts of DOX-SPIO/NPCS microparticle prepared under **(A,C,E)** formic acid, **(B,D,F)** formic acid/acetone solvent system.

**Figure 5:**
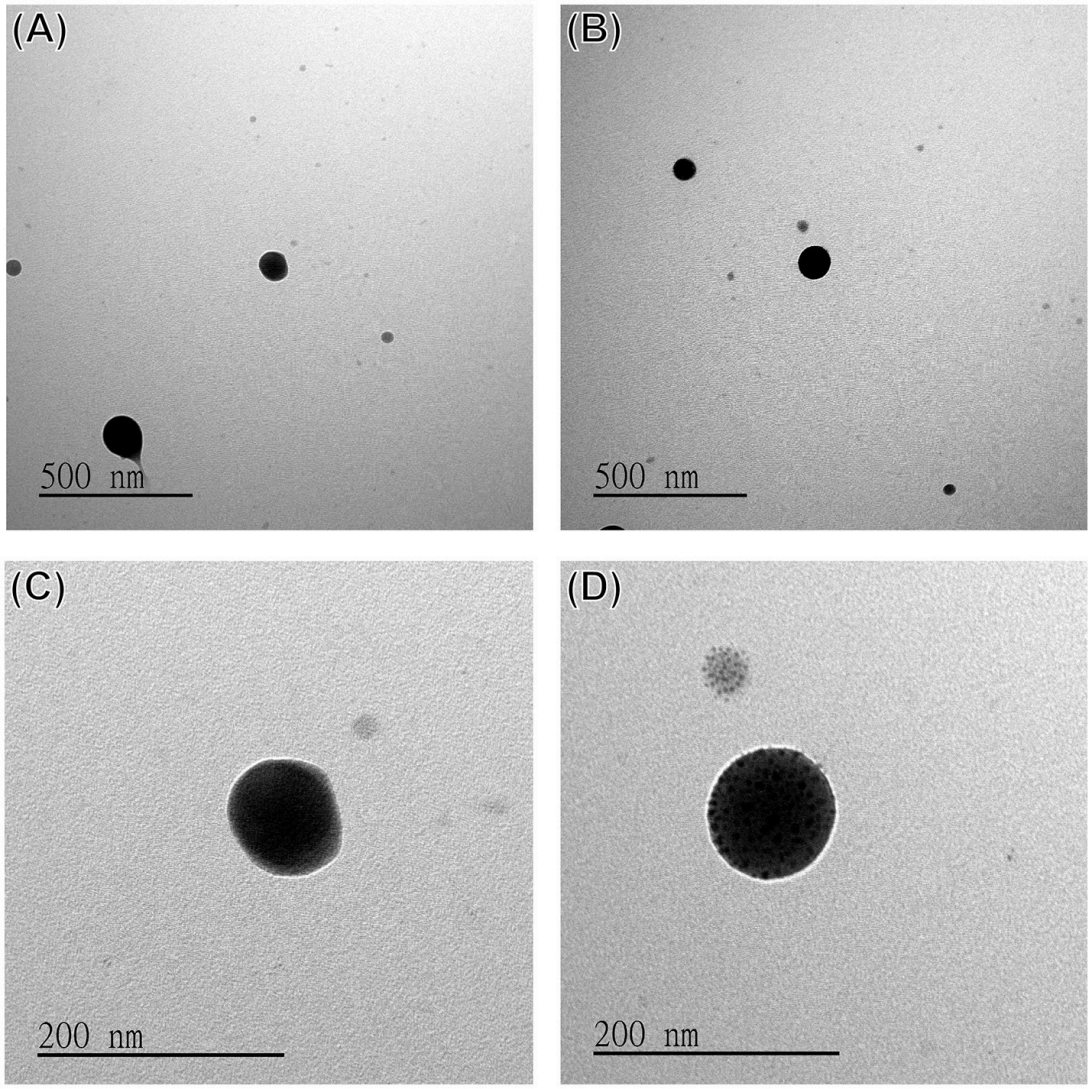
TEM images of **(A,C)** DOX/NPCS, **(B,D)** DOX-SPIO/NPCS microparticles prepared under parameters as follows: working distance 6.5 cm high, applied voltage ranged from 10 to 17 kV, and flow rate 2 μL/min.

The morphology of the DOX-SPIO/NPCS microparticles was observed in SEM images. All microparticles produced were spherical and uniform in shape, with sizes ranging from submicrometers to nanometers because different solvent systems resulted in different dielectric constants of the stock solution. As shown in Figure 4, the particle size produced in the formic acid/acetone solvent system (Figures 4B,D,F) fell within the range of 185 ± 87 nm, which was smaller than that produced in the pure formic acid solvent system (Figures 4A,C,E). This result demonstrates that particle sizes can be strongly influenced by the solvent system, when an ES technique was implemented in particle preparation. Solvents with high volatility and low viscosity properties could decrease the particle size significantly, and thus we used a formic acid/acetone cosolvent system for particle preparation. Although the dielectric constant of acetone (~20.24) is much lower than that of formic acid (58.5), the use of the formic acid/acetone cosolvent could strike a balance between viscosity and conductivity to generate DOX-SPIO/NPCS particles with more homogeneous and smaller sizes than those of the formic acid group. Because the penetration of an electron beam can be retarded by the heavy element of iron in SPIO, we were able to characterize the encapsulation of SPIO nanoparticles through TEM. Figures 5A–D show TEM images of the blank DOX/NPCS and DOX-SPIO/NPCS microparticles, respectively. Clustering of multiple dark points contributed by SPIO encapsulation was evident in the DOX-SPIO/NPCS microparticles. By contrast, vacant particles lacked dark clusters, as shown in the TEM images of the DOX-NPCS microparticles (Figures 5A,C).

### T2 relaxivity

**Figure 6:**
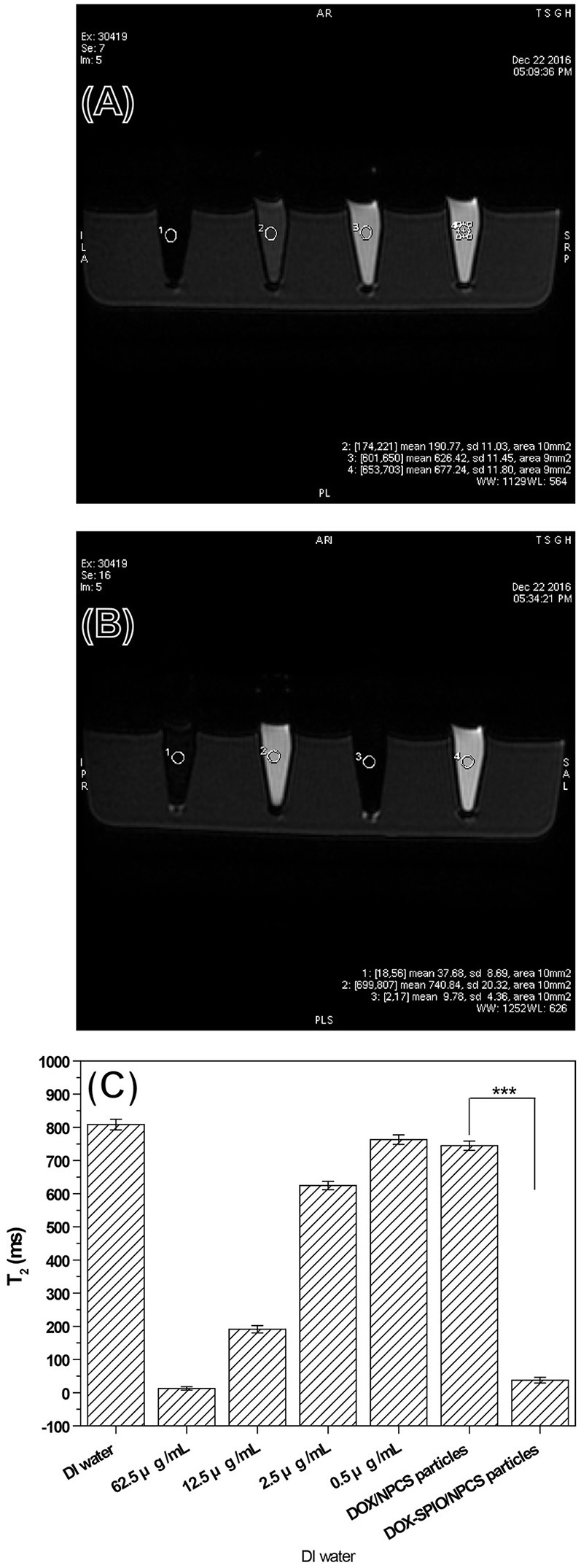
Signals of T2-weighted FSE MRI images from a series samples: **(A)** a series of DOX solution with concentration ranging from 0.5 to 62.5 μg/mL, **(B)** a series of DOX-SPIO/NPCS microparticle suspension with concentration ranging from 0 to 0.5 μg/mL and **(C)** quantatative value of T2 relaxation time.

The generation of negative contrast enhancement images is one of the key functions of visualizing varying hardness of tissue in clinical MRI practice. For example, T1 is strongly influenced by the mobility of the lattice; thus, in crystalline solids and viscous liquids, mobility is low and T1 is large. However, T2 is generally quite small for crystalline solids and viscous liquids. Thus, T2 images can be used as complementary images to differentiate soft tissue with different compositions or at different sites of the human body. A series of SPIO suspensions were studied *in vitro* at increasing concentrations and imaged on a 1.5-T GE MRI scanner and one-channel lower extremity coil to determine their T2-enhancing abilities. Images for measuring the iron concentration in serial dilutions were acquired using a sagittal two-dimensional FSE sequence. Our results indicated that the T2 signal decreased with increasing concentrations of SPIO from 0.5 to 62.5 μg/mL as the crystalline part content increased; this effect was dose-dependent (Figure 6A) and evidently attributable to the SPIO. To evaluate the potential for *in vitro* detection of DOX-SPIO/NPCS microparticles by using MRI in the T2 mode, four standard samples with or without SPIO encapsulation were prepared and imaged. To quantitatively estimate the reductions in the T2 signal in a series of SPIO and microparticle suspensions, a 10-mm2 area was labeled for each specimen test, as shown in Figure 6B. The pure SPIO suspension with a density of 62.5 μg/mL exhibited the lowest T2 signal (12.68 ± 5.01 ms). The DOX-NPCS microparticles and deionized water showed no significant reduction in the T2 spin-spin relaxation time (808.39 ± 16.06 and 744.63 ± 13.99 ms, respectively) because their mobility was quite high. However, after 2.5 μg/mL of SPIO was coincorporated into the NPCS carrier to generate the DOX-SPIO/NPCS microparticles, the T2 spin-spin relaxation time was significantly reduced (37.68 ± 8.69 ms) compared with that of the DOX-NPCS microparticles. Thanks to the confinement effect of the NPCS matrix, a remarkable reduction of spin-spin relaxation time was observed at a relatively low dosage of SPIO that was approximately comparable to the reduction at a pure SPIO concentration of 62.5 μg/mL. These results indicated that the encapsulation of SPIO in NPCS microparticles can provide one of the predetermined functions (i.e., T2-weighted MRI); in particular, the NPCS encapsulation matrix greatly increased the viscosity environment inside the microparticle, and thus significantly reduced the T2 spin-spin relaxation time at concentrations as low as 0.5 μg/mL to reveal a T2 value comparable to that of 62.5 μg/mL pure SPIO suspension; this result provides a great opportunity to reduce the dosage of SPIO in MRI imaging when necessary.

### *In vitro* release profile

**Figure 7:**
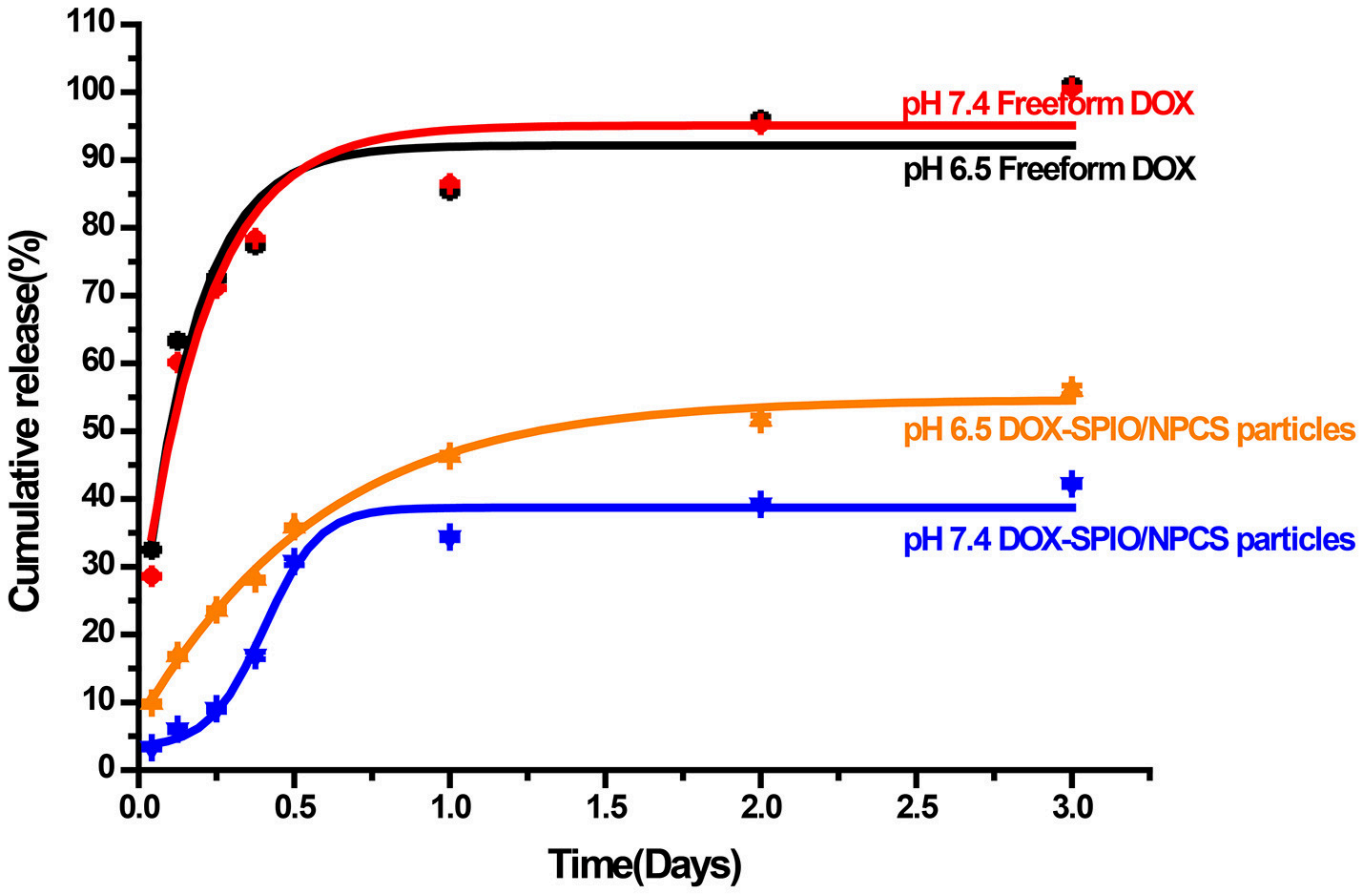
*In vitro* drug-release profiles of (•) DOX, and (▴) DOX-SPIO/NPCS microparticle soaked in PBS solution at pH 7.4 and 37°C for up to 72 h.

To investigate the DOX release behavior of DOX-SPIO/NPCS microparticles, these microparticles were incubated in multiple release media (pH 6.5 and 7.4) and assessed through ultraviolet (UV) spectrophotometer. Figure 7 shows an *in vitro* cumulative release profile of a suspension of DOX-SPIO/NPCS microparticles compared with that of an aqueous solution of free DOX. At the time point of 3 days, the cumulative release of free DOX was quantified as approximately 100% at a pH level of 6.5 or 7.4, whereas that of DOX encapsulated in the SPIO/NPCS carriers was reaching only 56.1% in an acidic environment and 39.2% in a basic environment. These results demonstrate that the relatively slow release rate in the basic medium and faster release rate in the acidic environment were attributable to the differently tortuous polymeric diffusion pathways in the encapsulating matrix material, whose tightness of structure can be deeply affected by pH changing. Consequently, such pH sensitivity could facilitate drug release within lower pH levels in targeted tumor regions. This feature enabled the DOX-SPIO/NPCS microparticles to not only prevent the harmful release of more than half of the DOX in healthy cells and tissues but also guarantee the effective release of a substantial quantity of the drug in the targeted area.

### Cytotoxicity

**Figure 8:**
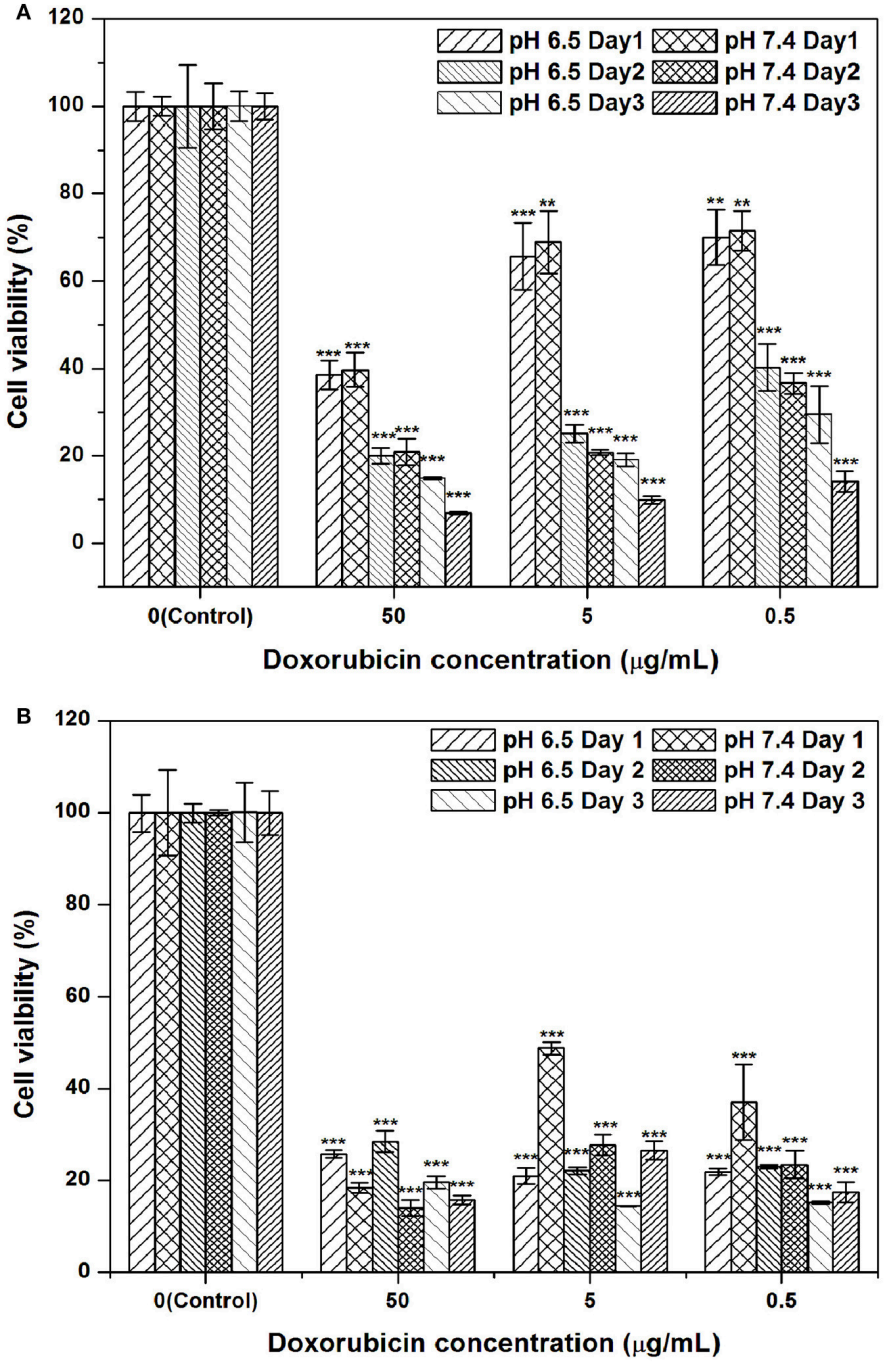
DOX-SPIO/NPCS microparticle suspension effectively suppressed hepatic cancer cell proliferation at concentrations of 0.5–50 μg/mL as compared to that of free form of DOX: **(A)** free form of DOX, and **(B)** DOX-SPIO/NPCS microparticle suspension. The same concentration of free DOX was also tested simultaneously for comparison. All cell viabilities shown in this figure are normalized to the negative control (NC) group treated with equivalent amounts of serum-free DMEM medium. *n* = 3 was tested for each group of trial. **p < 0.01 and ***p < 0.001, respectively.

An MTT assay was employed to assess the cytotoxicity of a series of formulations with multiple concentrations of DOX against the Hep G2 hepatic cancer cell line. The formulations dispersed in PBS at multiple concentrations were (1) free DOX solution and (2) DOX-SPIO/NPCS microparticles. Figure 8 shows the results of quantitative analyses of the cytotoxicity of the aforementioned formulations against Hep G2 compared with that of free DOX. The cell line was treated with multiple concentrations of DOX formulations (0.5, 5, and 50 μg/mL) for 24–72 h. Treatment with free DOX exerted dose- and time-dependent cytotoxic effects on the viability of the Hep G2 cells. Against our expectations, cells treated *in vitro* with the DOX-SPIO/NPCS microparticle formulation exerted a stronger cytotoxic effect on the viability of the Hep G2 cells as the expected releasing amount of DOX was approximately 1/2-1/3 of the total loading based on the *in vitro* release profile, and this effect can be enhanced in an acidic environment. Thus, DOX-SPIO/NPCS microparticle formulations can easily reach the half-maximal inhibitory concentration on the first day, even at a concentration as low as 0.5 μg/mL. Possible effects could be attributed to the protonation of NPCS in an acidic environment, which enabled the microparticles to be protonated (i.e., became -NH${}_{3}^{+}$) and expand in volume, thereby accelerating the drug release rate and their electrostatic interaction with the cell.

## Discussion

The ES technique was employed to generate microparticles because high-molecular-weight polymers (NPCS) were used as carriers to encapsulate multiple active ingredients (DOX and SPIO) without chemical reaction or use of abundant organic solvent. This technology is an electrohydrodynamic process for forming fine particles by applying a high voltage while liquid is spraying through a capillary and collection over a short distance with a ground electrode connected. Polymers as carriers have been reported in many studies alongside use of ES to synthesize drug-containing particles (Songsurang et al., 2011; Xu et al., 2013; Li et al., 2017). In our study, instead of using the conventional polymer, a pH-sensitive CS derivative (i.e., NPCS) was synthesized for application. When charged droplets fell into the collection substrate—a petri dish of distilled water—a bottle of DOX-SPIO/NPCS microparticle suspension was generated within a single step.

Based on our previous study results (Tang et al., 2017), we demonstrated that carriers composed of CS and chondroitin sulfate can incorporate DOX and SPIO simultaneously with use of ES technology in a single step. However, chondroitin sulfate may integrate with CS through ionic gelation before fluid spraying because of its electronegative property. Moreover, some sources of chondroitin sulfate come from cattle; this may lead to safety concerns related to Creutzfeldt–Jakob disease. Therefore, in the present study, chondroitin sulfate was replaced with NPCS, which is a material that possesses a pH response property in volume and is suitable for selective drug delivery at cancer sites.

NPCS is composed of a CS backbone and hydrophobic palmitoyl groups. CS with functionalization ability provided by the presence of amino and hydroxyl functional groups is regarded as a popular natural polymer. To render it pH sensitivity and improve drug delivery efficiency, NPCS is produced by conjugating a hydrophobic palmitoyl group onto the free amino groups of CS. At low pH, the free amino groups on CS grab external H^+^ to form protonated amine groups (−NH${}_{3}^{+}$), whose charge results in electrostatic repulsion, leading to the extension of polymer chains and volume. As the pH increases, the protonated amino groups on NPCS tend to be deprotonated (-NH_2_) and the hydrophobic side chains (palmitoyl groups) become dominated, causing NPCS to progressively condense because of the strong van der Waals force between long alkyl side chains (Chiu et al., 2009; Balan et al., 2015). In our study, we successfully synthesized NPCS according to the results of NMR spectroscopy, as shown in Figure 1. In addition, the swelling effect of NPCS varied with pH, as observed in Figure 2.

In a single step of electrospraying, the DOX-SPIO/NPCS microparticles with a spherical shape and 185 ± 87 nm in size were prepared as shown in Figure 4. The microparticles' composition and intended function were confirmed through a series of tests, including FTIR, TEM, encapsulation efficiency, MRI, and release experiments. The FTIR spectrum of the DOX-SPIO/NPCS microparticles (Figure 3F) showed a newly generated peak at 2,800–2,950 cm^−1^, which proved that the carrier matrix material was composed of N-palmitoyl-conjugated CS (i.e., NPCS). SPIO is verified by the dark points in the TEM images (Figure 5) and the declining T2 signal in the MRI experiments (Figure 6). Moreover, DOX can be detected at the maximum absorption peak by UV spectrophotometer at 480 nm in encapsulation efficiency and release experiments. The encapsulation efficiency of DOX in DOX-SPIO/NPCS microparticles was calculated using equation: EE(%) = W/W_0_ × 100%, in which EE is the encapsulation efficiency, W_0_ is the total amount of free drug loading, and W is the total amount of DOX encapsulated in the microparticles, respectively. Our study showed that the encapsulation efficiency of DOX was 26.6 ± 4.3% in DOX-SPIO/NPCS microparticles. These results are consistent with those of our previous study [8], thereby demonstrating that the ES technology is suitable for synthesizing multicomponent-loaded particles. As a major deviation from what was reported previously, this work focus on a development of pH-sensitive and volume-expandable microparticles, which is suitable for chemoembolization treatment.

TACE is a general therapeutic option for patients with unresectable HCC and is considered an alternative or bridging therapy before transplant surgery. However, only 35–40% of patients who undergo TACE can achieve an objective antitumor response (Bruix et al., 1998; Chan et al., 2002; Llovet et al., 2002). Moreover, a meta-analysis showed that tumor response with DEB-TACE, where drug-eluting beads are used with slowly releasing chemotherapeutic agents, was not significantly different from that with conventional TACE (Gao et al., 2013). Thus, post-TACE complications such as acute liver failure (7.5%) are thought to be responsible for precipitating ischemic damage not at the target site (Marelli et al., 2007). To improve antitumor response, combination therapy such as radiofrequency ablation or hyperthermia treatment plus TACE (Ni et al., 2013) and multifunctional embolization (yttrium-90-labeled microsphere) can induce extensive tumor necrosis and even lead to a higher survival rate than can TACE alone. In our case, the encapsulated SPIO has extra benefit to provide the function of hyperthermia treatment (see Figure S2 and Video 1) except the ability to show MRI T2 imaging in clinical use.

In summary, in the present study, we successfully prepared multifunctional, biocompatible, biodegradable, and highly antitumor response embolizing particles (DOX-SPIO/NPCS microparticles) that can theoretically generate many clinical benefits in the future application. For example, the vascular pore cutoff size of solid tumors is between 380 and 780 nm (Hobbs et al., 1998); hence, our particles with sizes of 185 ± 87 nm in neutral pH fall within the suitable range to pass through the leaky junction of the angiogenesis vessel into the tumor tissue according to the enhanced permeation and retention effect (EPR effect). Another benefit of using these large particles is that they are sufficiently large to obstruct vascular pores and even capillaries because of their swelling effect and aggregation behavior in acidic environment (Szymanska and Winnicka, 2015; Xiao et al., 2015). Thus, these particles combine the beneficial effects of traversing and obstructing small and big angiogenesis vessels to improve antitumor efficacy, which the treatment response can be simultaneously monitored by MRI T2 image.

## Conclusions

In this study, we developed a novel, multimodality, biodegradable DOX-SPIO/NPCS microparticle through a single-step ES process. We demonstrated the particles' potent ability of antitumor efficacy in the Hep G2 hepatic cancer cell line model and their slow release of DOX and sensitivity of T2 relaxation as MRI contrast agents. To the best of our knowledge, no studies of formulations of DOX, SPIO, and NPCS used as embolizing agents for hepatic cancer treatment have been conducted. This novel type of particle can simultaneously provide chemotherapy, embolization therapy, and MRI T2 image function at hepatic cancerous sites, and thus is expected to exhibit greater antitumor efficacy than conventional therapy. Our future studies will be aimed at preparing a complex of DOX, SPIO, and NPCS of larger size—between micrometers and millimeters—to study the efficacy of combining two sizes of embolizing particles and verifying it in an *in vivo* animal model.

## Author contributions

M-YB, M-HC, and S-LT designed and performed all experimental procedures, including setup of the electrospinning system, material preparation, and cell studies. S-LT helped with preparing the samples for magnetic resonance imaging experiment and provided medical materials and professional consultation about pharmaceutical use. M-YB, T-YW, and P-dH revised the manuscript critically for important intellectual content.

### Conflict of interest statement

The authors declare that the research was conducted in the absence of any commercial or financial relationships that could be construed as a potential conflict of interest.

## References

[B1] Ayuso C. Rimola J. Vilana R. Burrel M. Darnell A. García-Criado Á.. (2018). Diagnosis and staging of hepatocellular carcinoma (HCC): current guidelines. Eur. J. Radiol. 101, 72–81. 10.1016/j.ejrad.2018.01.025 29571804

[B2] Bai M. Y. Liu S. Z. (2014). A simple and general method for preparing antibody-PEG-PLGA sub-micron particles using electrospray technique: an *in vitro* study of targeted delivery of cisplatin to ovarian cancer cells. Colloids Surf. B Biointerf. 117, 346–353. 10.1016/j.colsurfb.2014.02.051 24681046

[B3] Bai M. Y. Yang H. C. (2013). Fabrication of novel niclosamide-suspension using an electrospray system to improve its therapeutic effects in ovarian cancer cells *in vitro*. Colloids Surf. A Physicochem. Eng. Aspects 419, 248–256. 10.1016/j.colsurfa.2012.11.076

[B4] Balan V. Dodi G. Tudorachi N. Ponta O. Simon V. Butnaru M. (2015). Doxorubicin-loaded magnetic nanocapsules based on N-palmitoyl chitosan and magnetite: synthesis and characterization. Chem. Eng. J. 279, 188–197. 10.1016/j.cej.2015.04.152

[B5] Bruix J. Llovet J. M. Castells A. Montañá X. Brú C. Ayuso M. D. C.. (1998). Transarterial embolization versus symptomatic treatment in patients with advanced hepatocellular carcinoma: results of a randomized, controlled trial in a single institution. Hepatology 27, 1578–1583. 10.1002/hep.510270617 9620330

[B6] Chan A. O. Yuen M. F. Hui C. K. Tso W. K. Lai C. L. (2002). A prospective study regarding the complications of transcatheter intraarterial lipiodol chemoembolization in patients with hepatocellular carcinoma. Cancer 94, 1747–1752. 10.1002/cncr.10407 11920537

[B7] Chen K. J. Chiu Y. L. Chen Y. M. Ho Y. C. Sung H. W. (2011). Intracellularly monitoring/imaging the release of doxorubicin from pH-responsive nanoparticles using Förster resonance energy transfer. Biomaterials 32, 2586–2592. 10.1016/j.biomaterials.2010.11.069 21251711

[B8] Chen Y. W. Liou G. G. Pan H. B. Tseng H. H. Hung Y. T. Chou C. P. (2015). Specific detection of CD133-positive tumor cells with iron oxide nanoparticles labeling using noninvasive molecular magnetic resonance imaging. Int. J. Nanomed. 10, 6997–7018. 10.2147/IJN.S86592 26635474 PMC4646596

[B9] Chiu Y. L. Chen S. C. Su C. J. Hsiao C. W. Chen Y. M. Chen H. L.. (2009). pH-triggered injectable hydrogels prepared from aqueous N-palmitoyl chitosan: *in vitro* characteristics and *in vivo* biocompatibility. Biomaterials 30, 4877–4888. 10.1016/j.biomaterials.2009.05.052 19527916

[B10] Cloupeau M. Prunet-Foch B. (1994). Electrohydrodynamic spraying functioning modes: a critical review. J. Aerosol. Sci. 25, 1021–1036. 10.1016/0021-8502(94)90199-6

[B11] Gao S. Yang Z. Zheng Z. Yao J. Deng M. Xie H.. (2013). Doxorubicin-eluting bead versus conventional TACE for unresectable hepatocellular carcinoma: a meta-analysis. Hepatogastroenterology 60, 813–820. 10.5754/hge121025 23282741

[B12] Hobbs S. K. Monsky W. L. Yuan F. Roberts W. G. Griffith L. Torchilin V. P.. (1998). Regulation of transport pathways in tumor vessels: role of tumor type and microenvironment. Proc. Natl. Acad. Sci. U.S.A. 95, 4607–4612. 10.1073/pnas.95.8.4607 9539785 PMC22537

[B13] Hsu C. Cheng J. Cheng A.-L. (2004). Recent advances in non-surgical treatment for advanced hepatocellular carcinoma. J. Formos. Med. Assoc. 103, 483–495. 15318270

[B14] Hu C.-S. Tang S.-L. Chiang C.-H. Hosseinkhani H. Hong P.-D. Yeh M.-K. (2014). Characterization and anti-tumor effects of chondroitin sulfate–chitosan nanoparticles delivery system. J. Nanoparticle Res. 16:2672 10.1007/s11051-014-2672-z

[B15] Jiang G.-B. Quan D. Liao K. Wang H. (2006). Novel polymer micelles prepared from chitosan grafted hydrophobic palmitoyl groups for drug delivery. Mol. Pharm. 3, 152–160. 10.1021/mp050010c 16579644

[B16] Jordan O. Denys A. De Baere T. Boulens N. Doelker E. (2010). Comparative study of chemoembolization loadable beads: *in vitro* drug release and physical properties of DC bead and hepasphere loaded with doxorubicin and irinotecan. J. Vasc. Interv. Radiol. 21, 1084–1090. 10.1016/j.jvir.2010.02.042 20610183

[B17] Lee Y. H. Mei F. Bai M. Y. Zhao S. Chen D. R. (2010). Release profile characteristics of biodegradable-polymer-coated drug particles fabricated by dual-capillary electrospray. J. Control. Release 145, 58–65. 10.1016/j.jconrel.2010.03.014 20346381

[B18] Li S. Xiao L. Deng H. Shi X. Cao Q. (2017). Remote controlled drug release from multi-functional Fe3O4/GO/Chitosan microspheres fabricated by an electrospray method. Colloids Surf. B Biointerf. 151, 354–362. 10.1016/j.colsurfb.2016.12.029 28043052

[B19] Liang G. Jia-Bi Z. Fei X. Bin N. (2007). Preparation, characterization and pharmacokinetics of N-palmitoyl chitosan anchored docetaxel liposomes. J. pharm. Pharmacol. 59, 661–667. 10.1211/jpp.59.5.0006 17524231

[B20] Lin C. K. Bai M. Y. Hu T. M. Wang Y. C. Chao T. K. Weng S. J.. (2016). Preclinical evaluation of a nanoformulated antihelminthic, niclosamide, in ovarian cancer. Oncotarget 7, 8993–9006. 10.18632/oncotarget.7113 26848771 PMC4891020

[B21] Liu Y. S. Lin X. Z. Tsai H. M. Tsai H. W. Chen G. C. Chen S. F.. (2015). Development of biodegradable radiopaque microsphere for arterial embolization-a pig study. World J. Radiol. 7, 212–219. 10.4329/wjr.v7.i8.212 26339465 PMC4553253

[B22] Llovet J. M. Real M. I. Montaña X. Planas R. Coll S. Aponte J.. (2002). Arterial embolisation or chemoembolisation versus symptomatic treatment in patients with unresectable hepatocellular carcinoma: a randomised controlled trial. Lancet 359, 1734–1739. 10.1016/S0140-6736(02)08649-X 12049862

[B23] Marelli L. Stigliano R. Triantos C. Senzolo M. Cholongitas E. Davies N. (2007). Transarterial therapy for hepatocellular carcinoma: which technique is more effective? A systematic review of cohort and randomized studies. Cardiovasc. Interv. Radiol. 30, 6–25. 10.1007/s00270-006-0062-3 17103105

[B24] Ni J. Y. Liu S. S. Xu L. F. Sun H. L. Chen Y. T. (2013). Transarterial chemoembolization combined with percutaneous radiofrequency ablation versus TACE and PRFA monotherapy in the treatment for hepatocellular carcinoma: a meta-analysis. J. Cancer Res. Clin. Oncol. 139, 653–659. 10.1007/s00432-012-1369-x 23292073 PMC11824270

[B25] Oliveri R. S. Wetterslev J. Gluud C. (2011). Transarterial (chemo) embolisation for unresectable hepatocellular carcinoma. Cochrane Database Syst. Rev. 16:CD004787 10.1002/14651858.CD004787.pub2 PMC12107519 21412886

[B26] Ramsey D. E. Geschwind J. F. H. (2002). New interventions for liver tumors. Semin. Roentgenol. 37, 303–11. 12455128 10.1016/s0037-198x(02)80007-4

[B27] Songsurang K. Praphairaksit N. Siraleartmukul K. Muangsin N. (2011). Electrospray fabrication of doxorubicin-chitosan-tripolyphosphate nanoparticles for delivery of doxorubicin. Arch. Pharm. Res. 34, 583–592. 10.1007/s12272-011-0408-5 21544723

[B28] Stubbs M. McSheehy P. M. Griffiths J. R. Bashford C. L. (2000). Causes and consequences of tumour acidity and implications for treatment. Mol. Med. Today 6, 15–19. 10.1016/S1357-4310(99)01615-9 10637570

[B29] Szymanska E. Winnicka K. (2015). Stability of chitosan—a challenge for pharmaceutical and biomedical applications. Mar. Drugs 13, 1819–1846. 10.3390/md13041819 25837983 PMC4413189

[B30] Tang S.-L. Bai M.-Y. Wang J.-Y. Hong P.-D. (2017). Development and application of micro-polysaccharide drug carriers incorporating doxorubicin and superparamagnetic iron oxide for bimodality treatment of hepatocellular carcinoma. Colloids Surf. B Biointerf. 151, 304–313. 10.1016/j.colsurfb.2016.12.036 28040662

[B31] Tapia-Hernndez J. A. Torres-Chvez P. I. Ramírez-Wong B. Rascn-Chu A. Plascencia-Jatomea M. Barreras-Urbina C. G. (2015). Micro-and nanoparticles by electrospray: advances and applications in foods. J. Agric. Food Chem. 63, 4699–4707. 10.1021/acs.jafc.5b01403 25938374

[B32] Weng L. Rostamzadeh P. Nooryshokry N. Le H. C. Golzarian J. (2013). *In vitro* and *in vivo* evaluation of biodegradable embolic microspheres with tunable anticancer drug release. Acta Biomater. 9, 6823–6833. 10.1016/j.actbio.2013.02.017 23419554

[B33] Xiao Y. Lin Z. T. Chen Y. Wang H. Deng Y. L. Le D. E.. (2015). High molecular weight chitosan derivative polymeric micelles encapsulating superparamagnetic iron oxide for tumor-targeted magnetic resonance imaging. Int. J. Nanomed. 10,1155–1172. 10.2147/IJN.S70022 25709439 PMC4330038

[B34] Xu S. Xu Q. Zhou J. Wang J. Zhang N. Zhang L. (2013). Preparation and characterization of folate-chitosan-gemcitabine core–shell nanoparticles for potential tumor-targeted drug delivery. J. Nanosci. Nanotechnol. 13, 129–138. 10.1166/jnn.2013.6794 23646707

[B35] Yeh M. K. Cheng K. M. Hu C. S. Huang Y. C. Young J. J. (2011). Novel protein-loaded chondroitin sulfate–chitosan nanoparticles: preparation and characterization. Acta Biomater. 7, 3804–3812. 10.1016/j.actbio.2011.06.026 21742066

