# Supplementary material for: Evaluation of Chitosan Derivative Microparticles Encapsulating Superparamagnetic Iron Oxide and Doxorubicin as a pH-Sensitive Delivery Carrier in Hepatic Carcinoma Treatment: An in vitro Comparison Study
Source: Front Pharmacol. 2018 Sep 21;9:1025. doi: 10.3389/fphar.2018.01025 (PMC6160595; doi:10.3389/fphar.2018.01025)
Supplement: Supplementary file 2 [file Table_1.DOCX]

**Supplementary materials**

**Evaluation of Chitosan Derivative Microparticles Encapsulating Superparamagnetic Iron Oxide and Doxorubicin as a pH-Sensitive Delivery Carrier in Hepatic Carcinoma Treatment: An In Vitro Comparison Study**

Meng-Yi Bai^1,2, 3^*, Sung-Ling Tang^1^, Meng-Han Chuang^2^, Ting-Ying Wang^4^, Po-da Hong^5^

^1^ Graduate Institute of Biomedical Engineering, National Taiwan University of Science and Technology, Taipei, Taiwan, Republic of China.

^2^ Biomedical Engineering Program, Graduate Institute of Applied Science and Technology, National Taiwan University of Science and Technology, Taipei, Taiwan, Republic of China.

^3^ Adjunct appointment to the Department of Biomedical Engineering, National Defense Medical Center, Taipei, Taiwan, Republic of China.

^4^ Department of Pharmacy Practice, Tri-Service General Hospital, Taipei, Taiwan, Republic of China.

^5^Department of Materials Science and Engineering, National Taiwan University of Science and Technology, Taipei, Taiwan, Republic of China.

^*^Corresponding author: Meng-Yi Bai, Graduate Institute of Biomedical Engineering and Biomedical Engineering Program, Graduate Institute of Applied Science and Technology, National Taiwan University of Science and Technology, 43 Keelung Road, Section 4, AAEON building, TR-917, Taipei, Taiwan, R.O.C.

Phone: +886-2-2730-3743

E-mail: mybai@mail.ntust.edu.tw

**Keywords:** nanotechnology, doxorubicin, superparamagnetic iron oxide, electrospray, microparticle.

**
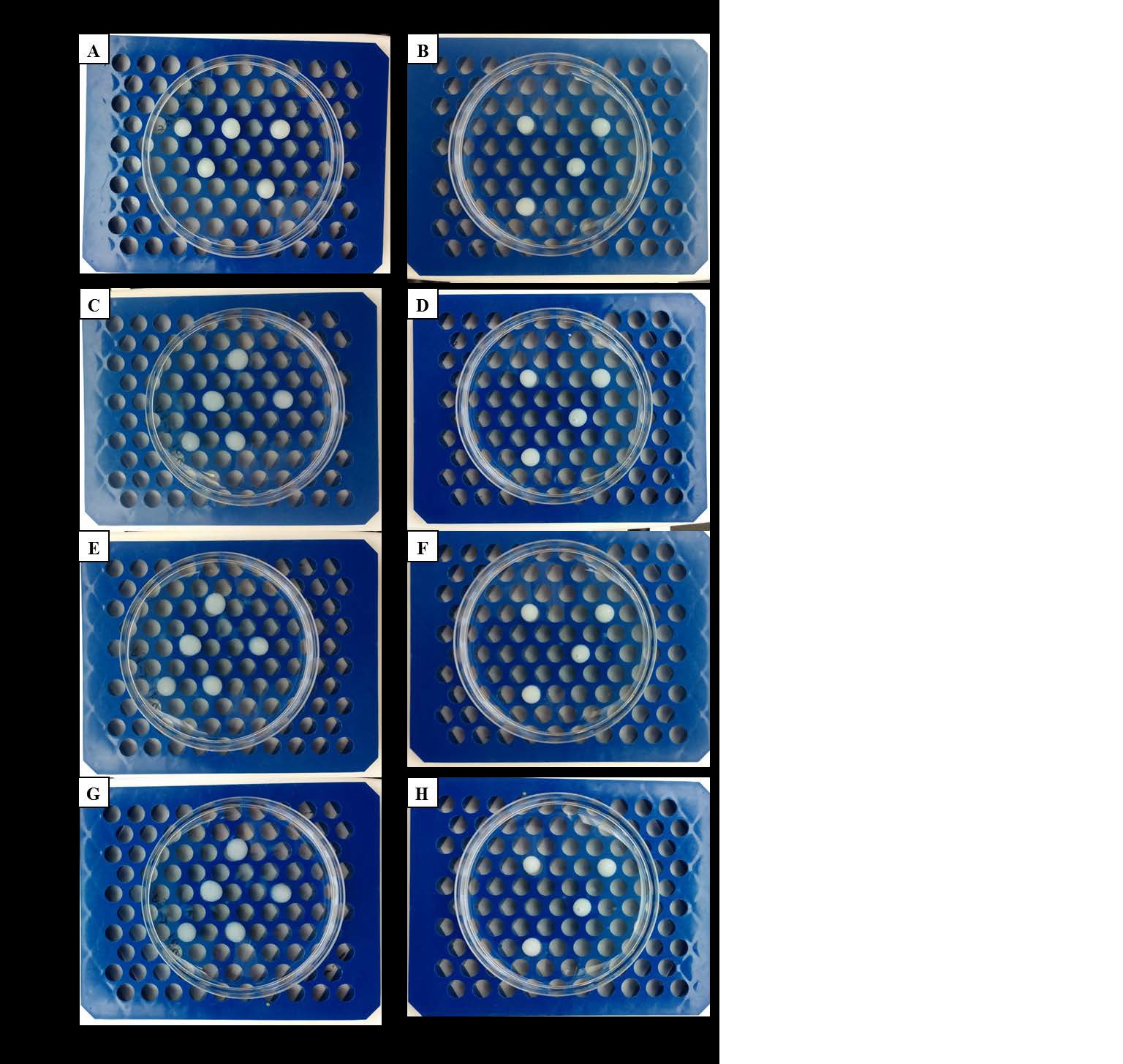
**

At pH= 6.5 At pH= 7.4

T= 0

T= 1 h

T= 2 h

T= 3h

**Fig. S1.** Photos of swelling test of NPCS at pH 6.5 and 7.4 from 0-3 hours.


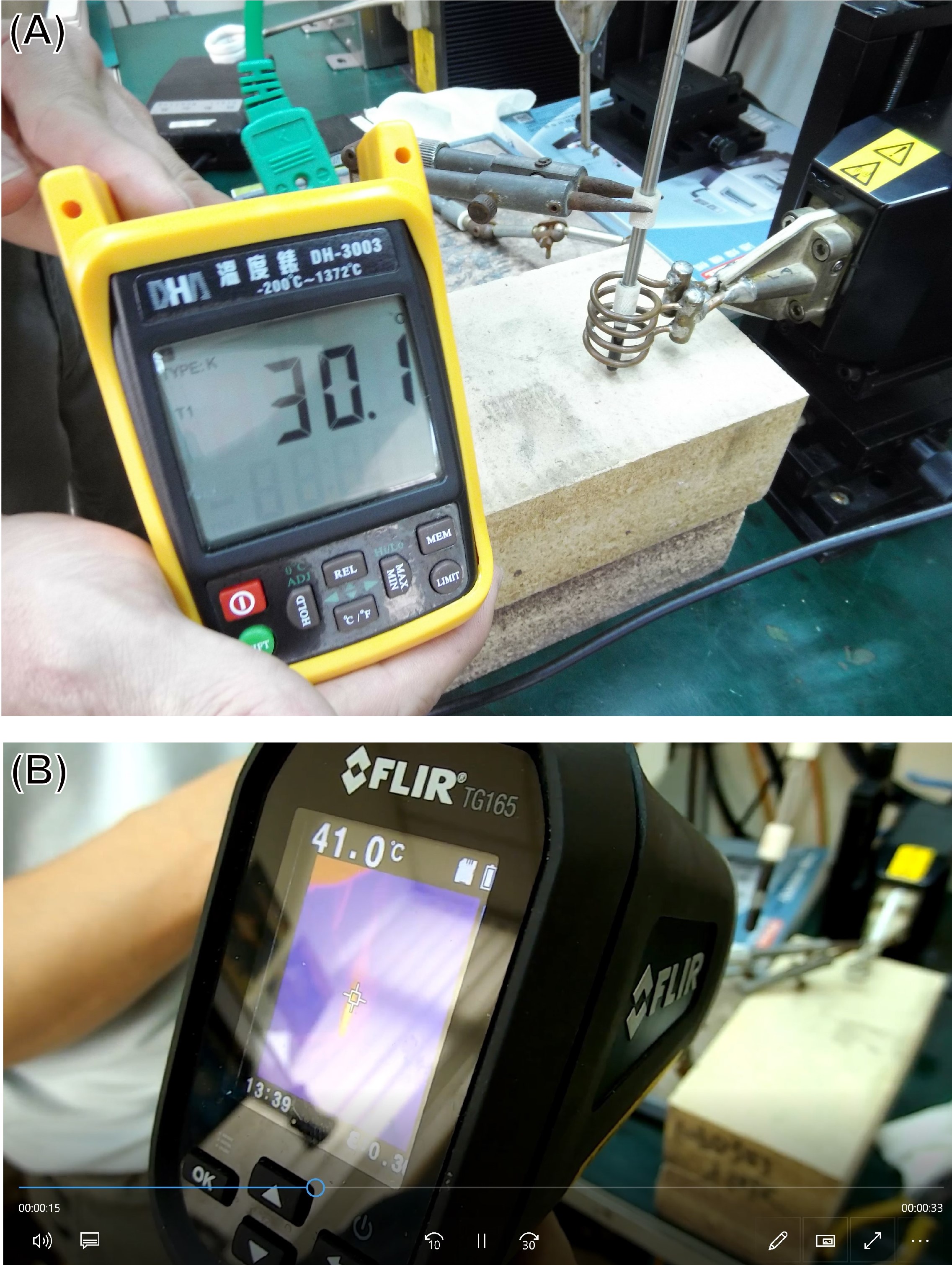


**Fig. S2.** Photos of various DOX-SPIO/NPCS microparticles after exposure under 20 sec, 50 w magnetic field oscillation: (A) DOX-SPIO/NPCS microparticles with encapsulation of 10 nm SPIO, temperature was raised from 25℃ to 30.1℃, (B) DOX-SPIO/NPCS microparticles with encapsulation of 10 μm SPIO, temperature was raised from 25℃ to 41.0℃. All samples were put in a 5 mm NMR tube and then be subject to a magnetic field oscillation for 20 sec.
